# Cytotoxicity, antibacterial efficacy, and fluoride content of commercial herbal toothpastes

**DOI:** 10.1038/s41405-026-00489-y

**Published:** 2026-10-01

**Authors:** Nuttanun Poeaim, Pasiree Thongthai, Pimduean Sivavong, Phakvalunch Rujiraprasert, Thanaphum Osathanon, Thawanrat Singthong, Budsaraporn Boonsuth, Dusit Nantanapiboon

**Affiliations:** 1 Department of Operative Dentistry, Faculty of Dentistry, Chulalongkorn University, Bangkok, Thailand; 2 Smart Innovations for Preventive and Restorative Dentistry Research Group, Faculty of Dentistry, Chulalongkorn University, Bangkok, Thailand; 3 Department of Cariology and Operative Dentistry, Graduate School of Medical and Dental Sciences, Institute of Science Tokyo, Tokyo, Japan; 4 Office of Research Affairs, Faculty of Dentistry, Chulalongkorn University, Bangkok, Thailand; 5 Center of Excellence for Dental Stem Cell Biology, and Department of Anatomy, Faculty of Dentistry, Chulalongkorn University, Bangkok, Thailand; 6 Dental Materials Research and Development Center, Faculty of Dentistry, Chulalongkorn University, Bangkok, Thailand; 7 Oral Biology Research Center, Faculty of Dentistry, Chulalongkorn University, Bangkok, Thailand; 8 Clinic of Gerodontology and Special Care Dentistry, Center for Dental Medicine, University of Zurich, Zurich, Switzerland; 9 Center of Excellence and Innovation for Oral Health and Healthy Longevity and Center of Excellence for Dental Stem Cell Biology, Faculty of Dentistry, Chulalongkorn University, Bangkok, Thailand

**Keywords:** Special care dentistry, Preventive dentistry

## Abstract

**Objective:**

This study evaluated fluoride concentration, antibacterial activity against Streptococcus mutans, and cytotoxicity of commercially available herbal toothpastes to assess their safety and clinical potential.

**Materials and methods:**

Fifteen herbal toothpastes were tested. Fluoride concentration was measured using a fluoride ion-selective electrode. Antibacterial activity against *S. mutans* was assessed by agar diffusion, and cytotoxicity was evaluated using the MTT assay in L929 fibroblasts and human gingival fibroblasts. Antibacterial and cytotoxicity data were analyzed using one-way ANOVA with Tukey’s HSD test, fluoride concentrations using the Kruskal–Wallis test, and associations among the three outcomes using Spearman’s rank correlation.

**Results:**

Fluoride concentrations varied considerably, with 66.67% of products containing less than 600 ppm and only 20% reaching approximately 1500 ppm. Most products did not clearly indicate fluoride type or concentration on their labels. Strong antibacterial activity, with inhibition zones of 2.12–2.18 cm comparable to vancomycin, was observed in 26.67% of samples (*p* < 0.05). LC₅₀ values ranged from 0.715 to 9.410, and four formulations showed high cytotoxicity (*p* < 0.05). Antibacterial activity was inversely correlated with the LC₅₀ of L929 cells (rs = −0.579, *p* < 0.001) and of human gingival fibroblasts (rs = −0.328, *p* < 0.05), whereas fluoride concentration showed no association with greater cytotoxicity.

**Conclusions:**

Herbal toothpastes with strong antibacterial effects often showed moderate to high cytotoxicity. Formulations containing biocompatible ingredients, antioxidants, and adequate fluoride may provide a safer balance, highlighting the need for clearer labeling and optimized ingredient concentrations.

## Introduction

Dental caries is currently understood as a multifactorial disease that is mediated by the oral biofilm and driven by dietary sugars, in which repeated cycles of demineralization and remineralization at the tooth surface become imbalanced and lead to a net loss of mineral from the dental hard tissues. The direction of this balance is modulated by host and environmental factors, among them saliva, tooth structure, oral hygiene practices, and fluoride exposure [1–3]. Within this biofilm, acidogenic and aciduric bacteria such as *Streptococcus mutans* metabolize dietary fermentable carbohydrates and produce organic acids that drive demineralization of the tooth enamel [2]. If this demineralization process is not prevented or reversed, repeated acid challenges promote progressive mineral loss, allowing the carious lesion to advance from the enamel into the dentin—a softer, more porous tissue beneath the enamel. Once the caries reaches the dentin, the decay process accelerates due to the higher organic content and lower mineral density of dentin compared to enamel. Eventually, caries can reach the pulp, causing infection and inflammation, which may lead to pain and require more extensive dental treatments [4]. The most effective and straightforward way to manage dental caries is through regular brushing with fluoride toothpaste. This simple practice can be easily incorporated into daily routines. Tooth brushing helps remove dental plaque, while fluoride in toothpaste enhances caries prevention, as supported by numerous systematic reviews [5–7].

In recent years, there has been increasing interest in organic oral healthcare products, driven by growing awareness of the potential adverse effects associated with certain chemical components commonly found in conventional dental care items, such as sodium lauryl sulfate (SLS), triclosan, preservatives (e.g., parabens), and synthetic colorants such as tartrazine (CI 19140) [8–13]. In an ASEAN country, the use of organic herbs in daily life, particularly in healthcare products like toothpaste and mouth rinse, is becoming increasingly popular, as evidenced by the variety of products and ingredients in children’s toothpastes commercially available in Thailand, as highlighted in the research by Thongthai et al. [14, 15]. As a result, patients are paying more attention to products made from natural and herbal extracts, especially when it comes to toothpaste and mouth rinse [16].

Herbal toothpaste has been used for generations in various forms, such as gel, paste, or powder, to remove plaque, prevent gum disease, and improve oral health [17]. Common organic oral healthcare ingredients such as clove, cinnamon, coconut, lemon, and spearmint oils are valued for their pain-relieving and antimicrobial properties [18]. Guava leaf extract is noted for its anti-inflammatory [19] and antibacterial effects [20]. Additionally, camphor is widely recognized for its soothing and cooling benefits [21, 22]. Many also contain natural abrasives like salt or bamboo charcoal, as well as turmeric [23, 24] and licorice [25, 26] for their anti-inflammatory and antimicrobial qualities. These natural ingredients are increasingly incorporated into dental products for their therapeutic effects on oral health.

Research on the toxicity of herbal toothpaste yielded mixed results. Some studies suggested that certain herbal ingredients might cause mild irritation or allergic reactions in sensitive individuals, while others found no significant adverse effects [27, 28]. The variability in these results could have been due to differences in the formulations of herbal toothpastes, the concentration of active ingredients, or the methodologies used in the studies [28, 29]. For example, essential oil–derived components frequently used in herbal dentifrices, including eugenol (clove oil) and menthol, have been reported to reduce the viability of oral fibroblasts in a concentration-dependent manner and may therefore cause tissue irritation if not appropriately formulated [28, 30], and borneol has been reported to inhibit the proliferation of primary oral fibroblasts in a dose-dependent manner, without cytotoxicity at the lower concentrations tested [31]. Conversely, other research showed that these same ingredients, when used in appropriate concentrations, were safe and effective for oral health [16, 32].

Overall, existing research has not reached a definitive conclusion regarding the toxicity of herbal toothpastes, emphasizing the need for further studies to thoroughly evaluate the safety and efficacy of these products [33]. Although many herbal toothpastes are available in the Thai market, there has been no research evaluating their fluoride content, effects on oral cell viability, or bacterial growth inhibition. Toothpaste is regulated in Thailand as a cosmetic product, for which the total fluoride concentration must not exceed 1500 ppm; at the time this study was conducted, however, manufacturers were not required by law to declare the fluoride concentration on the product label [34]. This study evaluates the fluoride concentration, antibacterial efficacy against *Streptococcus mutans*, and cytotoxicity on L929 and human gingival fibroblast cells of herbal toothpastes, to support their clinical relevance. The null hypothesis was that there would be no differences among the tested herbal toothpastes in fluoride concentration, antibacterial activity against *S. mutans*, or cytotoxicity.

## Materials and methods

### Herbal toothpastes preparation

Approval was obtained from the ethics committee (HREC-DCU 2022-074). Fifteen commercially available toothpastes marketed as herbal or natural, or containing herbal-derived ingredients, were included in this study. Each product was purchased from a retail store, a pharmacy, or an official online retailer in Thailand. Products were selected to represent a broad range of herbal toothpaste formulations commercially available in Thailand, including fluoride-free and fluoride-containing formulations, different fluoride compounds, and a variety of herbal ingredients. Product names, lot numbers, expiration dates, and the compositions reported by the manufacturers are presented in Table 1.

**Table 1:** Overview and composition of 15 selected commercially available herbal toothpastes.

Product lists	Product names	Lot number and expiration date	Manufacture composition
1	Tree Herb Natural Herbal Extract Toothpaste	Lot number 001B 220424 Expiration date 22-04-2027	Siamese rough bush (Toothbrush tree), guava leaf extract, clove, borneol, and camphor.
2	Thipniyom Herbal Toothpaste	Lot number 03 09032024 Expiration date 09-03-2027	Calcium Carbonate, Water, Sorbitol, Glycerin, Sodium Lauryl Sulfate, Carboxymethyl Cellulose, Menthol Crystals, Hydrophilic Pyrogenic Silica, Sodium bicarbonate, Peppermint Oil, Clove Oil, Borneol Flakes, TR-1 Compound, Sage Oil, Myrrh Oil, Camphor, Camomile Blue Oil.
3	Moa Jula Herbal Toothpaste	Lot number 10 080224 Expiration date 08-02-2027	Water, Dicalcium Phosphate Dihydrate, Sorbitol, Sodium Lauryl Sulfate, Sodium Chloride, Glycerin, Silica, *Mentha piperita* Oil, Cellulose Gum, Camphor, Methylparaben, Propylparaben, *Eugenia caryophyllus* Flower Oil, *Cinnamomum cassia* Leaf Oil, Aluminum Sulfate, Borneol.
4	Dentiste 100% Natural Toothpaste	Lot number 4003290524 Expiration date 29-05-2027	Sorbitol, Hydrated Silica, Purified Water, Propanediol, Sodium Lauroyl Lactylate, Sodium Bicarbonate, *Mentha Piperita* Oil, Xanthan Gum Kaolin, Xylitol, *Stevia Rebaudiana* Extract, *Beta Vulgaris* Extract, *Psidium Guajava* Extract, *Eucalyptus Globulus* Leaf Oil, Glyceryl Caprylate, Glyceryl Undecylenate, *Foeniculum Vulgare* Extract, *Glycyrrhiza Glabra* Extract, *Cinnamomum Cassia Bark* Extract, *Salvia Officinalis* Extract.
5	Kolbadent	Lot number 100524 105 Expiration date 10-05-2027	Glycerin, Sorbitol, Purified Water, Dicalcium Phosphate, *Streblus asper* Leaf Extract, *Eugenia Caryophyllus* Leaf Oil, Mint.
6	Colgate Naturals Panjaved	Lot number 08/19 B16 CP Expiration date 07-2025	Calcium Carbonate, Water, Sorbitol, Sodium Lauryl Sulfate, Hydrated Silica, Zinc Oxide, Sodium Silicate, Carrageenan, Sodium Monofluorophosphate, *Eugenia Caryophyllus* Oil, Flavor, Zinc Citrate, Sodium Bicarbonate, *Mentha Arvensis* Oil, Benzyl Alcohol, Potassium Nitrate, Sodium Saccharin, CI 74160, Camphor, *Emblica Officinalis* Fruit Extract, CI 77491, *Azadirachta Indica* Seed Oil, CI 74160, *Foeniculum Vulgare* Oil, Honey Extract, *Aloe Barbadensis* Leaf Extract, *Cinnamomum Zeylanicum* Leaf Oil, *Ocimum Basilicum* Oil.
7	Tepthai Concentrated Herbal Toothpaste	Lot number E0225062426 Expiration date 15-06-2027	Calcium Carbonate, Water, Sorbitol, Sodium Lauryl Sulfate, Sodium Carboxymethyl Cellulose, Borneol, Menthol, Camphor, *Streblus asper* Leaf Extract, Guava Extract, Licorice Extract, Clove Oil, *Eclipta prostrata* Extract, Peppermint Extract.
8	Goodage Herbal Total Care	Lot number 170124 2 Expiration date 17-01-2027	Sorbitol, water, Hydrated Silica, Sodium Chloride, Propylene Glycol, Cellulose Gum, Flavor, PEG-20 Hydrogenated Castor Oil, Sodium Cocoyl Glutamate, Sodium Lauryl Sulfate, Sodium Fluoride, CI 77891, Sodium Saccharin, Sodium Hydroxide, Maltodextrin, Ethylparaben, *Phyllanthus Emblica* fruit Extract, *Terminalia Bellerica* fruit Extract, Butylparaben, CI 19140, CI 17200, CI 42090.
9	Salz Triphala	Lot number 140823 3 Expiration date 14-08-2025	Sorbitol, water, Hydrated Silica, Sodium Chloride, Sodium Lauryl Sulfate, Propylene Glycol, Flavor, PEG-20 Hydrogenated Castor oil, Cellulose Gum, CI 77891, Sodium Hydroxide, Sodium Fluoride, Sodium Saccharin, Dipotassium Glycyrrhizate, Maltodextrin, Ethylparaben, *Phyllanthus Emblica* Fruit Extract, *Terminalia Bellerica* Fruit Extract, *Terminalia Chebulan* Fruit Extract, Butylparaben, CI 19140, CI 17200, CI 42090.
10	Darlie Salt Herbal Protect	Lot number 20221011HH414 13212 Expiration date 09-10-2025	Water, Calcium Carbonate, Sorbitol, Sodium Chloride, Sodium Lauryl Sulfate, Hydrated Silica, Flavor, PEG-12, Poloxamer 407, Cellulose Gum, Sodium Monofluorophosphate, Sodium Carbonate, Sodium Saccharin, Xanthan Gum, Hydrated Silica/CI 74260, Sodium Bicarbonate, CI 47005, CI 42051.
11	Parodontax Herbal Fresh	Lot number 01032024 Expiration date 01-03-2027	Sodium Bicarbonate, Aqua, Glycerin, Sodium Lauryl Sulfate, Aroma, Xanthan Gum, Cocamidopropyl Betaine, Sodium Fluoride, Titanium Dioxide, Steviol Glycosides (Stevioside, Rebaudioside A), CI 77492, CI 174160. Sodium Fluoride 0.221% w/w (1000 ppm fluoride).
12	Dok Bua Ku Original Toothpaste	Lot number 0124225 Expiration date 04-07-2029	Sorbitol (Natural Origin from Cassava), Calcium Carbonate (Mineral Abrasive), Cuttlefish Bone, Toothbrush Tree, *Clinacanthus nutans*, Sodium Lauryl Sulfate (Natural Origin from Coconut Oil), Hydrated Silica (Mineral Thickener), Orange Jessamine, Peppermint Oil, Menthol, Eucalyptus Oil, Sodium Benzoate.
13	Veldent Nature Boost Oriental Mint Flavor	Lot number 3010 131223 Expiration date 13-12-2026	Sorbitol, water, Silica, Sodium Lauryl Sulfate, Cellulose Gum, Titanium Dioxide, Sodium Benzoate, Menthol, Potassium Sorbate, Menthyl Succinate, Sodium Saccharin, Xylitol, Maltodextrin, Tocopheryl Acetate, Calcium Lactate, *Malpighia Glabra* Fruit Extract, Butylene Glycol, *Chamomilla Recutita* Extract, Ubiquinone, *Equisetum Arvense* Extract, Ascorbic Acid, Sodium Citrate, Citric Acid, *Glycyrrhiza Glabra* Root Extract, Phenoxyethanol, Cyanocobalamin, Cetyl Palmitate, C12-15 Alkyl Benzoate, Glycerin, Lauryl Glucoside, Xanthan Gum, Flavor, CI 42090, CI 19140.
14	Hi-Herb Day & Night Care	Lot number 170124 1 Expiration date 17-01-2027	Water, Sorbitol, Hydrated Silica, Propylene Glycol, Sodium Lauryl Sulfate, Flavor, Cellulose Gum, Sodium Silicate, CI 77891, Sodium Fluoride, Zinc Lactate, Sodium Saccharin, Menthol, Methylparaben, *Eugenia Caryophyllus* Flower Oil, Dipotassium Glycyrrhizate, o-Cymen-5-ol, *Philodendron Amurense* Bark Extract, Glycerin, Butylparaben, *Camellia Sinensis* Leaf Extract, *Psidium Guajava* Leaf Extract, CI 42090, *Chamomilla Recutita* Flower Extract, *Mentha Piperita* Leaf Extract, *Rosmarinus Officinalis* Leaf Extract, *Salvia Officinalis* Leaf Extract.
15	CUdent Triple Herb Toothpaste	Lot number K001151121 Expiration date 15-05-2024	Aqua (Water), Calcium Carbonate, Sorbitol, Glycerin, Hydrated Silica, Tetrasodium Pyrophosphate, Cellulose Gum, Sodium Monofluorophosphate, Sodium Lauroyl Sarcosinate, Aroma (Flavor), Sodium Benzoate, Rebaudioside A, Xylitol, Benzyl Alcohol, Eugenol, Menthol, *Aloe Barbadensis* Leaf Juice Powder, *Eugenia Caryophyllus* (Clove) Bud Oil.

Toothpaste solutions were prepared following ISO 11609: 2017 standards. Specifically, 1 g of each toothpaste sample was dissolved in 3 mL of deionized water and thoroughly mixed. Subsequently, 3 mL of the resulting mixture was transferred to centrifuge tubes and subjected to centrifugation at 4000 rpm for 15 min. The supernatant was carefully collected and immediately utilized for subsequent analyses.

### Determination of total fluoride content

The total fluoride concentration was determined using a fluoride ion-selective electrode according to previously described methods, with modifications [14, 35]. Total Ionic Strength Adjustment Buffer (TISAB IV) and 70% perchloric acid (Merck, Darmstadt, Germany) were used for sample preparation. TISAB IV was prepared according to the manufacturer’s instructions. Quality-control fluoride solutions were prepared at concentrations of 1000, 500, 250, 125, and 62.5 ppm to verify the accuracy and consistency of the fluoride measurements. Fifteen commercially available herbal toothpaste were selected for analysis, with the preparation of each sample following the method previously described. The fluoride concentration was measured by mixing 1 ml of the prepared toothpaste solution with 1 ml of TISAB IV. The fluoride ion-selective electrode (Versa Star; Thermo Fisher Scientific Inc., Waltham, MA, USA) was calibrated using external fluoride standards at concentrations of 1, 10, 100, and 1000 ppm before sample measurement. Each measurement was conducted three times to ensure reproducibility, and the mean values of fluoride concentration (ppm F) were calculated. These findings were subsequently compared with the fluoride content listed by the manufacturers.

An adapted procedure was applied to toothpaste containing sodium monofluorophosphate. For toothpastes containing sodium monofluorophosphate (MFP), an additional acid-hydrolysis step was performed according to a previously described protocol [14]. In MFP, fluoride is bound as the monofluorophosphate ion (FPO_3_^2−^), which is not detected by the fluoride ion-selective electrode; acid hydrolysis is therefore required to cleave the P–F bond and release fluoride as free F^−^ before measurement. Acid hydrolysis of MFP prior to fluoride determination is an established step in fluoride-analysis protocols [35, 36]. Briefly, a 4 mL aliquot of the prepared toothpaste solution was mixed with 1 mL of 70% perchloric acid and maintained at room temperature for 24 h before fluoride analysis.

### Antibacterial efficacy

Antibacterial activity against *Streptococcus mutans* was evaluated using an agar diffusion assay based on previously described methods [14, 37, 38]. *Streptococcus mutans* (ATCC 25175) from a –80 °C stock was cultured on sheep blood agar (SBA) and incubated at 37 °C with 5% CO_2_ for 20–24 h. A single colony was then transferred to 5 mL of Mueller Hinton Broth (MHB; HIMEDIA, India) and incubated under the same conditions for 24 hours. The bacterial suspension was adjusted to a turbidity of 0.5 McFarland (equivalent to 1.0 × 10^8 CFU/mL), and the optical density was measured at a wavelength of 600 nm using a spectrophotometer. A 50 μL aliquot of the adjusted bacterial suspension was spread onto Mueller-Hinton Agar (MHA; HIMEDIA, India) with 5% sheep blood using a sterile cotton swab, ensuring the swab was spread to the edge of the agar plate. A 6-mm-diameter paper disk was soaked with 20 μL of the prepared toothpaste solution, while deionized water served as the negative control, and a Vancomycin Antimicrobial Susceptibility disk (OXOID, Hampshire, UK) was used as the positive control. Each disk was gently pressed on the agar surface to ensure even contact. The disks were carefully spaced a minimum of 24 mm apart and placed on a 100-mm Petri dish. The disks were carefully positioned to remain stationary and set at an appropriate distance from the agar plate’s edge, ensuring no disruption to the inhibition zone for accurate antibacterial measurements. The plates were incubated at 37 °C with 5% CO_2_ for 24 h. The antibacterial assay was performed as a single experiment in which three replicate disks were tested for each toothpaste formulation and for each control (*n* = 3 replicates per group). Antibacterial activity was evaluated by measuring the diameter of the complete inhibition zone using a digital Vernier caliper.

### Cell culture for cytotoxicity assay

Human gingival fibroblast (HGF) cells were isolated from viable gingival tissues collected from healthy volunteers aged 20–60 years undergoing routine dental procedures at the Department of Oral Surgery, Faculty of Dentistry, Chulalongkorn University. The protocol was adapted from Cvikl et al. (2015) [39]. Fresh tissue samples were immersed in Dulbecco’s Modified Eagle Medium (DMEM) supplemented with 10% Antibiotic-Antimycotic solution and 1% L-glutamine (GlutaMAX). Under sterile conditions, tissues were sectioned into ~0.5 × 0.5 cm² fragments and placed in new culture dishes. Complete growth medium (85% DMEM, 10% fetal bovine serum (FBS), 1% GlutaMAX, 3% Antibiotic-Antimycotic, and 1% Fungizone) was added carefully to prevent desiccation or floating. Cultures were maintained at 37 °C in a humidified incubator with 5% CO₂. Fibroblast outgrowth was typically observed within 5–7 days. Tissue remnants were removed, and the medium was changed every three days. Cells were expanded and sub-cultured at 80–90% confluency for cytotoxicity testing. The L929 mouse fibroblast cell line (NCTC clone 929, CCL-1, ATCC; Lot No. 70026472) was cultured in Eagle’s Minimum Essential Medium (EMEM) supplemented with 10% FBS, 1% GlutaMAX, 3% Antibiotic-Antimycotic, and 1% Fungizone. Cells were maintained at 37 °C with 5% CO₂ and passaged upon reaching confluency, following ISO 10993-5:2009 guidelines.

After passaging, 100 µl of culture medium containing 3 × 10^4^ cells were seeded into 96-well plates and incubated under the same conditions. After 24 h, the medium was removed, and the cells were rinsed with 100 µl of phosphate-buffered saline (PBS). Toothpaste solutions were prepared as previously described but were diluted with culture media instead of distilled water. Each well was then treated with 100 µl of the toothpaste solution and incubated for 2 min before assessing cell viability. A control was included, consisting of a culture medium without toothpaste. The MTT solution (3-[4,5-dimethylthiazol-2-yl]-2,5-diphenyl tetrazolium bromide) was prepared by dissolving 1 mg of MTT in 1 ml of DMEM without supplements or phenol red. A 50 µl aliquot of the MTT solution was added to each well, and the plate was incubated in a humidified atmosphere with 5% CO_2_ at 37 °C for 2 hours. After incubation, the MTT solution was discarded, and 100 µl of DMSO was added to each well to dissolve the formazan crystals. The plates were gently shaken to ensure thorough dissolution, and the resulting solution was transferred to a new well plate. Absorbance at 570 nm was measured using a UV spectrophotometer, and cell viability was calculated as a percentage relative to the control. Each toothpaste concentration was tested in three replicate wells within a single experiment (*n* = 3), and the LC50 was determined separately for each well.

### Statistical analysis

Statistical analysis was performed using IBM SPSS Statistics 26 (SPSS, Chicago, IL, USA). Zone of inhibition data were compared among products using one-way analysis of variance (ANOVA) followed by Tukey’s honestly significant difference (HSD) test, with a significance threshold set at *p* < 0.01. LC50 values for L929 cells and human gingival fibroblasts were compared using the same procedure, with significance set at *p* < 0.05. Differences among products in fluoride concentration were assessed using the Kruskal–Wallis test. Associations among fluoride concentration, zone of inhibition and LC50 in both cell types were examined using Spearman’s rank correlation; coefficients are reported with their exact two-tailed *p* values.

### Ethics approval and consent to participate

This experiment was approved by the Human Research Ethics Committee of the Faculty of Dentistry, Chulalongkorn University (Study code: HREC-DCU 2022-074), reviewed and approved the procedure on September 9th, 2022.

## Results

### Measurement of total fluoride concentration

**Fig. 1:**
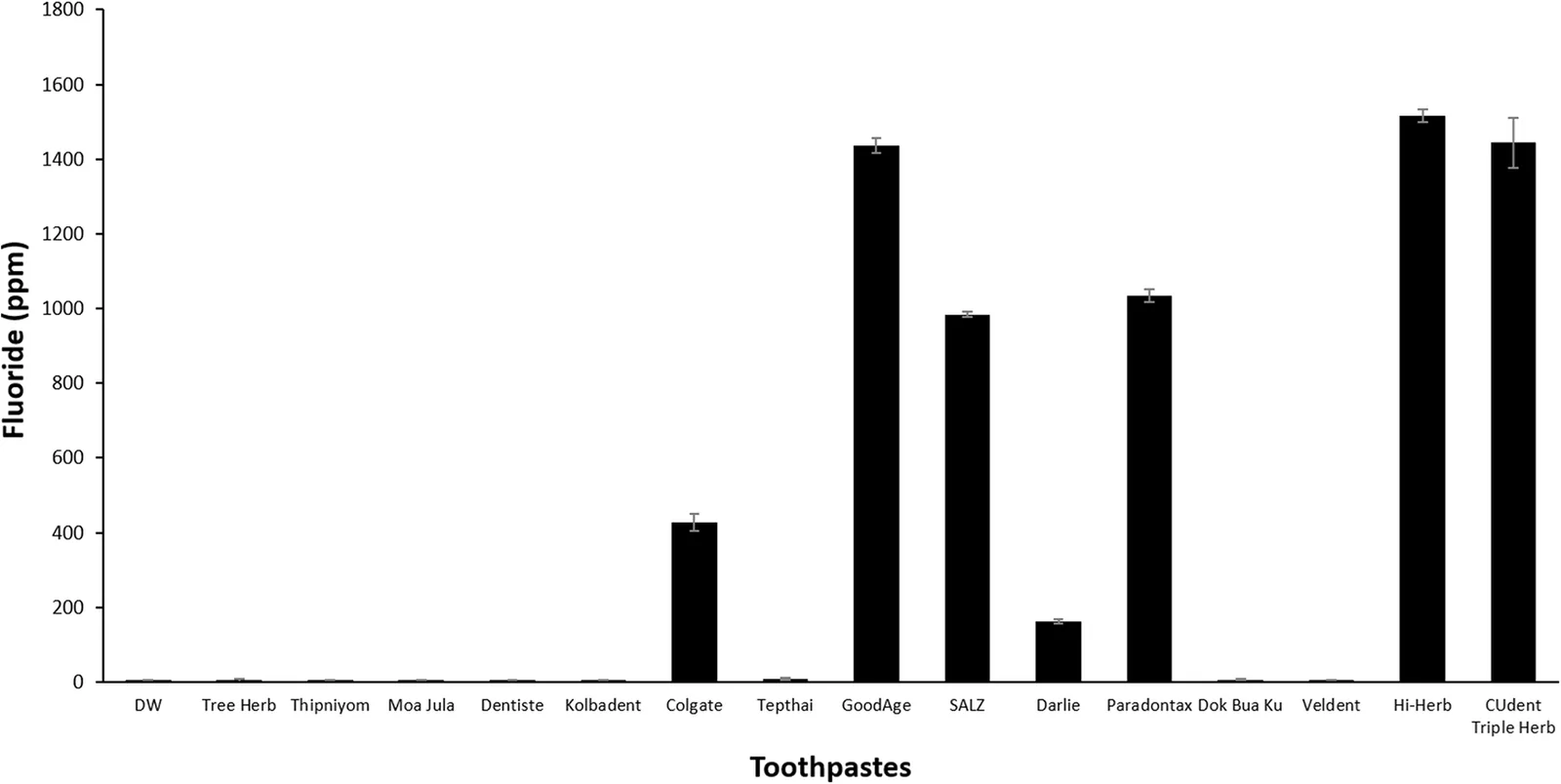
Fluoride ion concentration (F^-^) determined in commercial herbal toothpastes (mean, *n* = 3). Error bars indicate SD.

All fluoride concentration results are presented in Fig. 1. Most of the herbal toothpastes tested lacked fluoride or had fluoride levels below 600 ppm (10 out of 15 samples: 66.67%), while the others contained either sodium fluoride (4 out of 15 samples: 26.67%) or sodium monofluorophosphate (3 out of 15 samples: 20.00%). Among the products tested, Hi-Herb Day & Night Care had the highest fluoride concentration at 1515.82 ± 18.35 ppm, followed by CUdent Triple Herb at 1444.06 ± 66.96 ppm, and Goodage Herbal Total Care at 1436.58 ± 20.26 ppm, all within the range of 1500 ppm. Additionally, Parodontax Herbal Fresh showed a fluoride concentration of 1034.70 ± 19.36 ppm, and Salz Triphala had 984.43 ± 6.61 ppm, both within the range of 1000 ppm. Colgate Naturals Panjaved, on the other hand, revealed a concentration of 427.70 ± 16.38 ppm, within the 500-ppm range. However, the other herbal toothpastes in this experiment showed fluoride concentrations similar to that of distilled water. Fluoride concentration differed significantly among the fifteen products (H(14) = 39.80, *p* < 0.001). Table 2 presents a comparison between the fluoride content listed on the product labels and the fluoride concentration determined through measurement with a fluoride ion-selective electrode. Most of the herbal toothpastes did not list fluoride on their product labels, and some did not indicate the fluoride concentration. However, Goodage Herbal Total Care and CUdent Triple Herb showed similar fluoride concentrations when comparing the product label information with the measurements obtained using a fluoride ion-selective electrode.

**Table 2:** Fluoride-ion (F^-^) concentration determined in fifteen commercial herbal toothpastes with standard deviation (*n* = 3), fluoride types, and the amount of fluoride certification from the manufacturers.

Samples	Herbal toothpastes names	Fluoride types/ Fluoride certification (ppm F)	Fluoride concentration (mean ± SD) (ppm F)
	Distilled water	None/ 0	4.16 ± 0.14
1	Tree Herb Natural Herbal Extract	None/ 0	4.10 ± 3.69
2	Thipniyom Herbal	None/ 0	5.65 ± 0.65
3	Moa Jula Herbal	None/ 0	6.43 ± 0.31
4	Dentiste 100% Natural	None/ 0	4.93 ± 0.14
5	Kolbadent	None/ 0	5.00 ± 0.12
6	Colgate Naturals Panjaved	Sodium Monofluorophosphate/ Not indicated	427.70 ± 16.38
7	Tepthai Concentrated Herbal	None/ 0	7.89 ± 2.85
8	Goodage Herbal Total Care	Sodium Fluoride/ 1500	1436.58 ± 20.26
9	Salz Triphala	Sodium Fluoride/ Not indicated	984.43 ± 6.61
10	Darlie Salt Herbal Protect	Sodium Monofluorophosphate/ Not indicated	161.15 ± 6.66
11	Parodontax Herbal Fresh	Sodium Fluoride/ 1000	1034.70 ± 19.36
12	Dok Bua Ku Original	None/ 0	4.87 ± 2.69
13	Veldent Nature Boost Oriental Mint Flavor	None/ 0	8.06 ± 0.31
14	Hi-Herb Day & Night Care	Sodium Fluoride/ Not indicated	1515.82 ± 18.35
15	CUdent Triple Herb	Sodium Monofluorophosphate/ 1498	1444.06 ± 66.96

ppm F: part per million Fluoride.

### Antibacterial efficacy

**Fig. 2:**
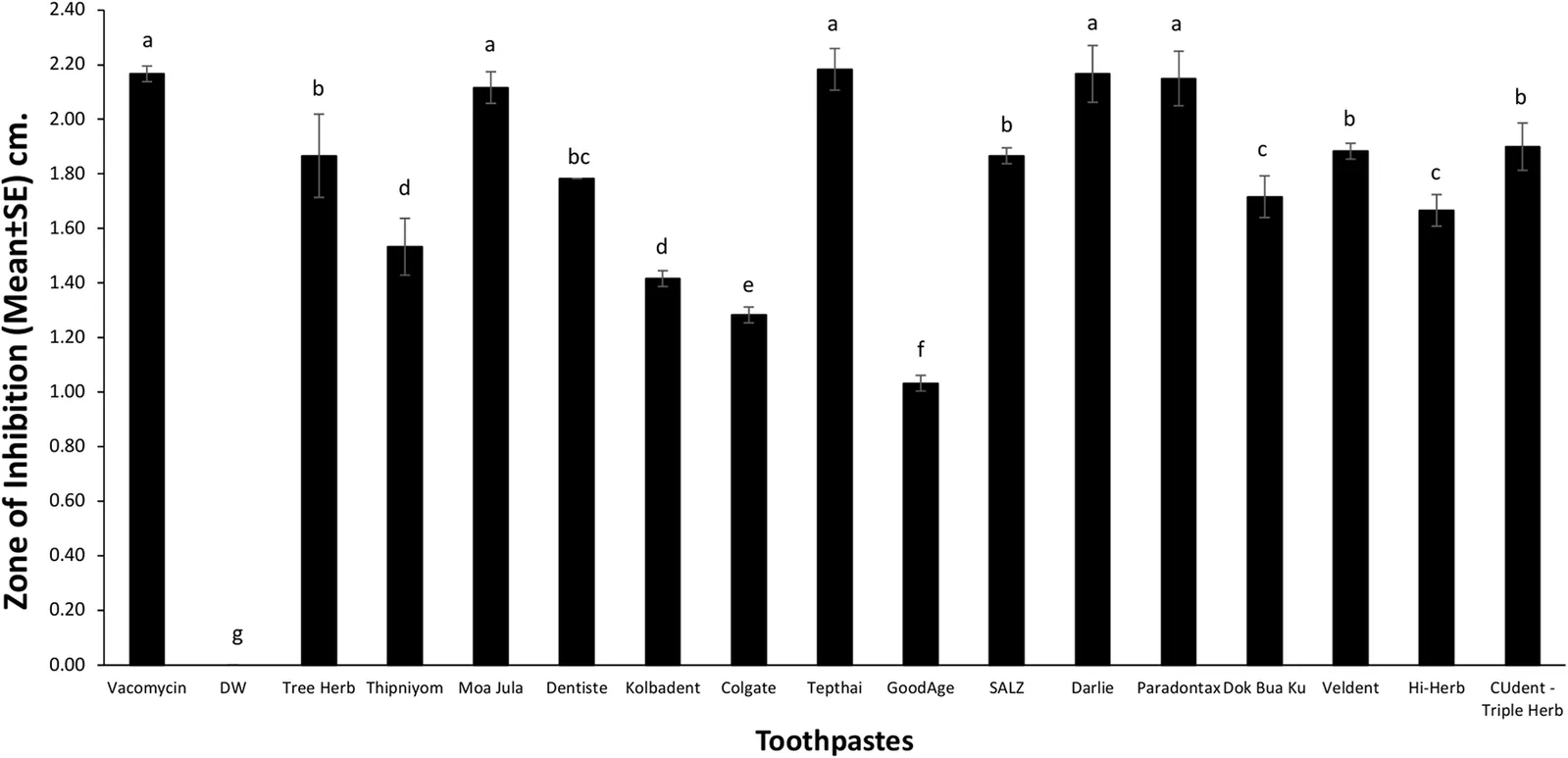
Zone of inhibition of fifteen commercial herbal toothpastes against *Streptococcus mutans*. The bar represents the standard deviation. **a**–**g** No significant differences between the same letters (analysis of variance [ANOVA], Tukey’s honestly significant difference [HSD] test, *p* < 0.01, *n* = 3).

The diameter of the inhibition zones for *S. mutans* in all tested herbal toothpastes is shown in Fig. 2 and reported in Table 3. In this study, a Vancomycin antimicrobial susceptibility disc served as a positive control, demonstrating an inhibition zone of 2.17 ± 0.03 cm. Distilled water was used as a negative control, showing no inhibition zone (0.00 ± 0 cm). Among the herbal toothpastes, Tepthai Concentrated Herbal Toothpaste, Darlie Salt Herbal Protect, Parodontax Herbal Fresh, and Moa Jula Herbal Toothpaste exhibited the highest antibacterial activity against cariogenic bacteria, with inhibition zones measuring 2.18 ± 0.08 cm, 2.17 ± 0.10 cm, 2.15 ± 0.10 cm, and 2.12 ± 0.06 cm, respectively. These results were statistically significant (*p* < 0.05) compared to other brands. The antibacterial efficacy of these four herbal toothpastes was comparable to that of the control, Vancomycin 30 µg, which is known for its effectiveness against gram-positive bacteria (*p* < 0.05). In contrast, Goodage Herbal Total Care showed the lowest antibacterial activity against cariogenic bacteria, with an inhibition zone of 1.03 ± 0.03 cm, which was also statistically significant when compared to the other toothpaste brands (*p* < 0.05).

**Table 3:** Mean and standard deviation (SD, *n* = 3) of the zone of inhibition of fifteen commercial herbal toothpastes against *Streptococcus mutans*.

Herbal toothpastes names	Zone of inhibition (Mean ± SD) cm.
Vancomycin	2.17 ± 0.03^a^
Distilled water	0.00 ± 0.00 ^g^
Tree Herb Natural Herbal Extract	1.87 ± 0.15^b^
Thipniyom Herbal	1.53 ± 0.10 ^d^
Moa Jula Herbal	2.12 ± 0.06^a^
Dentiste 100% Natural	1.78 ± 0.03^bc^
Kolbadent	1.42 ± 0.03 ^d^
Colgate Naturals Panjaved	1.28 ± 0.03^e^
Tepthai Concentrated Herbal	2.18 ± 0.08^a^
Goodage Herbal Total Care	1.03 ± 0.03 ^f^
Salz Triphala	1.87 ± 0.03^b^
Darlie Salt Herbal Protect	2.17 ± 0.10^a^
Parodontax Herbal Fresh	2.15 ± 0.10^a^
Dok Bua Ku Original	1.72 ± 0.08^c^
Veldent Nature Boost Oriental Mint Flavor	1.88 ± 0.03^b^
Hi-Herb Day & Night Care	1.67 ± 0.06^c^
CUdent Triple Herb	1.90 ± 0.09^b^

### Cytotoxicity assay

**Fig. 3:**
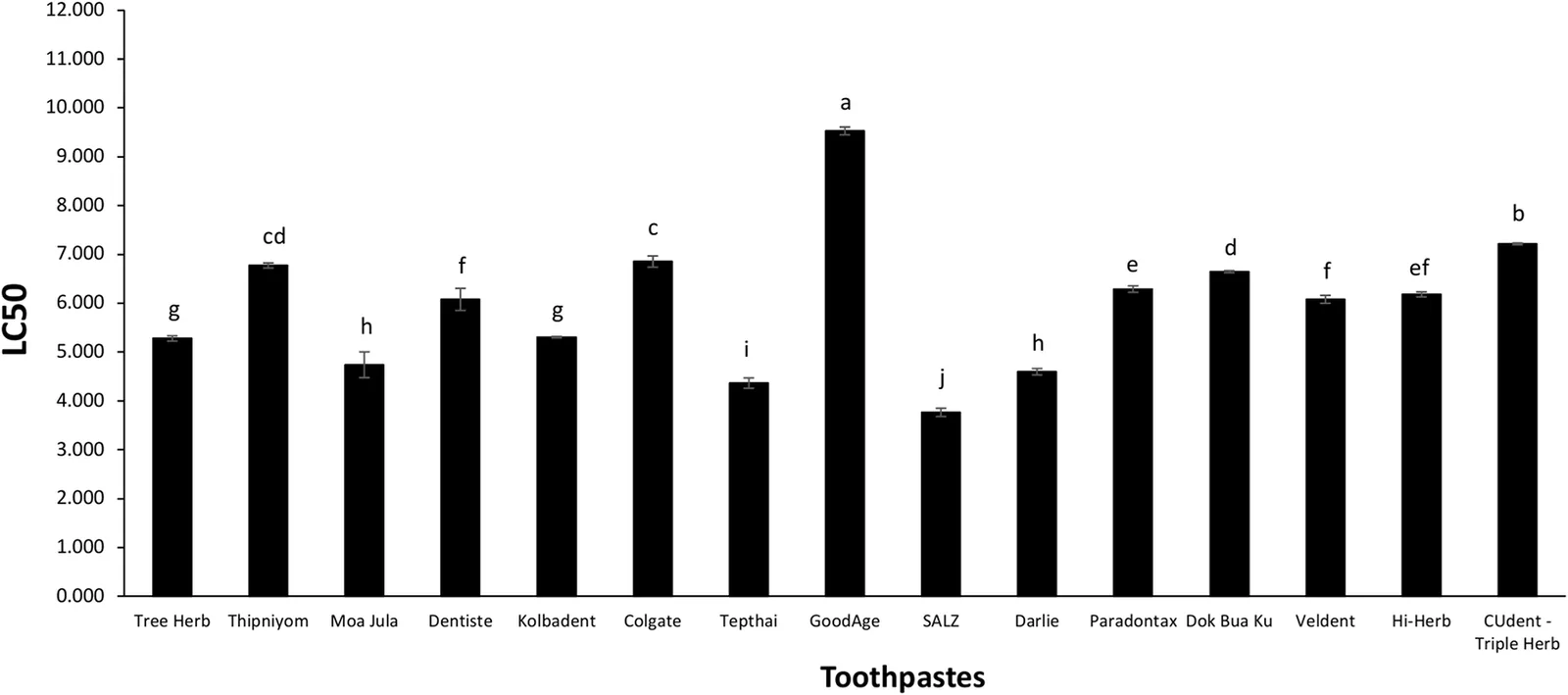
50% lethal concentration (LC_50_) of L929 cells was measured by the MTT assay after a 2-minute exposure to different concentrations of commercial herbal toothpastes. The bars represent the standard deviation. **a**–**j** No significant differences between the same letters (analysis of variance [ANOVA], Tukey’s honestly significant difference [HSD] test, *p* < 0.05, *n* = 3).

**Fig. 4:**
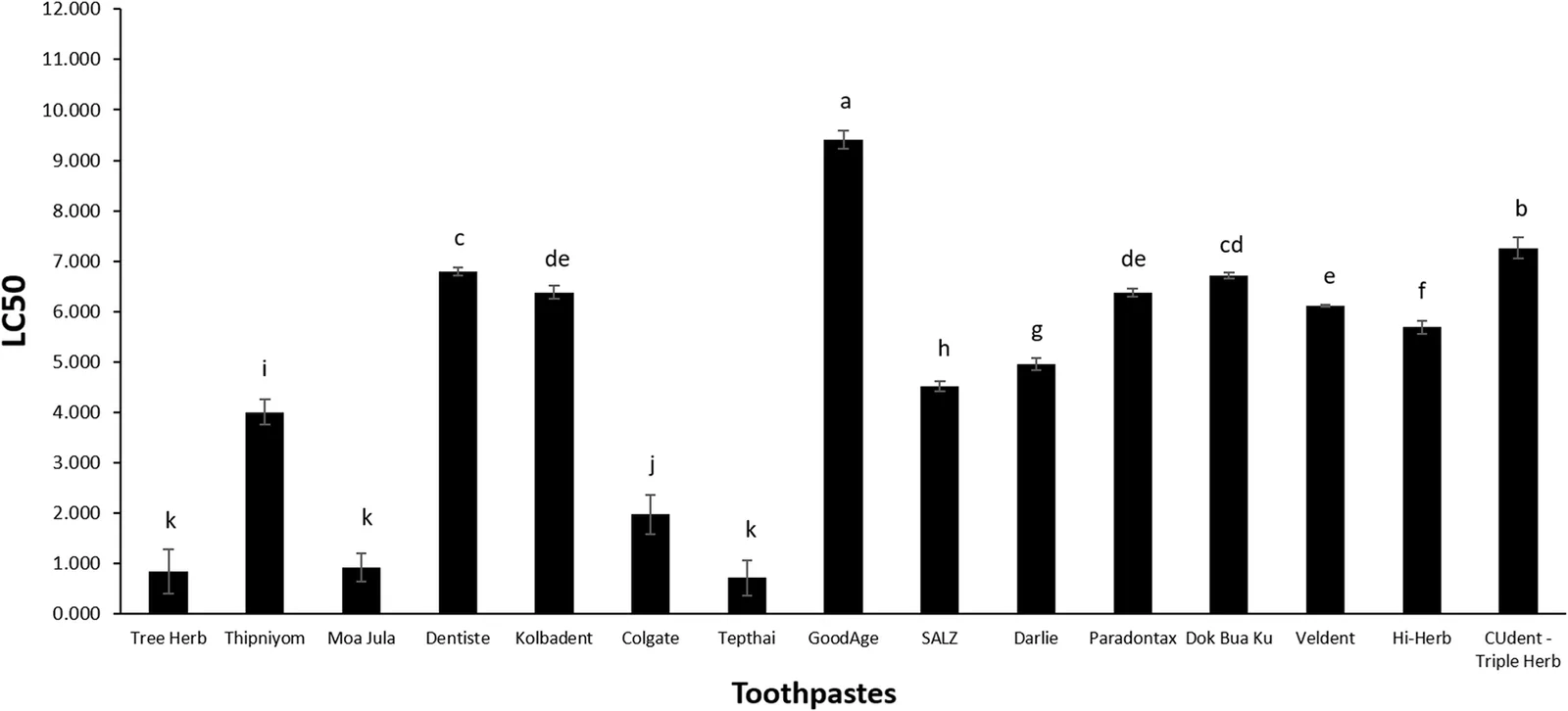
50% lethal concentration (LC_50_) of HGFs cells was measured by the MTT assay after a 2-minute exposure to different concentrations of commercial herbal toothpastes. The bars represent the standard deviation. **a**–**j** No significant differences between the same letters (analysis of variance [ANOVA], Tukey’s honestly significant difference [HSD] test, *p* < 0.05, *n* = 3).

The cytotoxicity test on L929 cells revealed that Salz Triphala exhibited the lowest LC₅₀ value (3.767 ± 0.084), indicating the highest cytotoxicity. This was followed by Tepthai Concentrated Herbal Toothpaste (4.366 ± 0.105), Darlie Salt Herbal Protect (4.598 ± 0.066), and Moa Jula Herbal Toothpaste (4.740 ± 0.263), all of which were significantly different (*p* < 0.05). In contrast, Goodage Herbal Total Care demonstrated the highest LC₅₀ value (9.530 ± 0.081), indicating the lowest cytotoxicity (*p* < 0.05), as shown in Fig. 3. A subsequent test using HGF cells (Fig. 4) confirmed similar findings. Tepthai Concentrated Herbal Toothpaste, Tree Herb Natural Herbal Extract Toothpaste, and Moa Jula Herbal Toothpaste showed the lowest LC₅₀ values, 0.715 ± 0.346, 0.841 ± 0.431, and 0.917 ± 0.281, respectively, indicating high cytotoxicity. Colgate Naturals Panjaved followed with an LC₅₀ of 1.972 ± 0.395. Goodage Herbal Total Care again showed the highest LC₅₀ value (9.410 ± 0.174), reflecting the lowest cytotoxicity (*p* < 0.05). A comparison of LC₅₀ values in both L929 and HGF cells is summarized in Table 4.

**Table 4:** 50% lethal concentration (LC_50_) of L929 cells and HGF cells were measured by the MTT assay after a 2-min exposure to different concentrations of commercial herbal toothpastes.

Herbal toothpastes names	LC_50_ (100% of control) (*n* = 3) ± SD	
	L929 cells	HGF cells
Tree Herb Natural Herbal Extract	5.278 ± 0.054 ^g^	0.841 ± 0.431^k^
Thipniyom Herbal	6.773 ± 0.051 ^cd^	4.007 ± 0.246^i^
Moa Jula Herbal	4.740 ± 0.263 ^h^	0.917 ± 0.281^k^
Dentiste 100% Natural	6.079 ± 0.227 ^f^	6.794 ± 0.090^c^
Kolbadent	5.306 ± 0.015 ^g^	6.384 ± 0.135^de^
Colgate Naturals Panjaved	6.857 ± 0.114^c^	1.972 ± 0.395^j^
Tepthai Concentrated Herbal	4.366 ± 0.105^i^	0.715 ± 0.346^k^
Goodage Herbal Total Care	9.530 ± 0.081^a^	9.410 ± 0.174^a^
Salz Triphala	3.767 ± 0.084^j^	4.524 ± 0.102 ^h^
Darlie Salt Herbal Protect	4.598 ± 0.066 ^h^	4.961 ± 0.116 ^g^
Parodontax Herbal Fresh	6.291 ± 0.065^e^	6.381 ± 0.080^de^
Dok Bua Ku Original	6.647 ± 0.024 ^d^	6.710 ± 0.066 ^cd^
Veldent Nature Boost Oriental Mint Flavor	6.082 ± 0.082 ^f^	6.115 ± 0.022^e^
Hi-Herb Day & Night Care	6.182 ± 0.052^ef^	5.689 ± 0.125 ^f^
CUdent Triple Herb	7.216 ± 0.016^b^	7.259 ± 0.210^b^

*LC*_50_ lethal concentration, *L929* mouse fibroblast cell line, *HGF* human gingival fibroblast.

### Correlation among fluoride concentration, antibacterial activity and cytotoxicity

Spearman’s rank correlation was used to examine the relationships among the three outcomes (Table 5). Antibacterial activity was inversely correlated with the LC50 of both cell lines, that is, toothpastes producing larger inhibition zones were more cytotoxic (L929, rs =  −0.579, *p* < 0.001; HGF, rs = −0.328, *p* = 0.028). The LC50 values of the two cell lines were positively correlated with each other (rs = 0.577, *p* < 0.001). Fluoride concentration was not correlated with antibacterial activity (rs = −0.007, *p* = 0.964), and showed weak positive correlations with the LC50 of L929 cells (rs = 0.322, *p* = 0.031) and of human gingival fibroblasts (rs = 0.267, *p* = 0.076), indicating that a higher fluoride content was not associated with greater cytotoxicity. Because a higher LC50 denotes lower cytotoxicity, a negative coefficient in Table 5 indicates an association with greater cytotoxicity.

**Table 5:** Pairwise correlations among fluoride concentration, antibacterial activity against Streptococcus mutans, and cytotoxicity in L929 cells and human gingival fibroblasts.

Variables	Fluoride concentration	Zone of inhibition	LC50 (L929)	LC50 (HGF)
Fluoride concentration	1.000			
Zone of inhibition	−0.007 (*p* = 0.964)	1.000		
LC50 (L929)	0.322 (*p* = 0.031)	−0.579 (*p* < 0.001)	1.000	
LC50 (HGF)	0.267 (*p* = 0.076)	−0.328 (*p* = 0.028)	0.577 (*p* < 0.001)	1.000

Values are Spearman’s rank correlation coefficients with two-tailed *p* values in parentheses (*n* = 45). A higher LC50 indicates lower cytotoxicity; a negative coefficient with an LC50 therefore indicates an association with greater cytotoxicity.

## Discussion

This study evaluated the fluoride concentration, antibacterial activity against *S. mutans*, and cytotoxicity of fifteen commercially available herbal toothpastes. Considerable differences were observed among the tested products in all three outcomes. Most products contained low fluoride concentrations, whereas antibacterial activity and cytotoxicity varied substantially among formulations. Therefore, the null hypothesis was rejected.

Herbal toothpaste is a dental product formulated with plant-based or naturally derived ingredients, typically excluding synthetic chemicals and therapeutic concentrations of pharmaceutical agents [16]. These products are increasingly favored for their perceived safety, minimal side effects, and environmental sustainability. Commonly used botanical ingredients include neem, clove, cinnamon, coconut, lemon, spearmint oils, and guava leaf extract—each recognized for their bioactive properties that promote oral health [18]. For example, guava (*Psidium guajava*) leaf extract is noted for its anti-inflammatory [19] and antibacterial activities [20], which may contribute to the prevention of oral diseases such as gingivitis.

Patients who prefer herbal products often believe these products are safer than those containing synthetic chemicals. This perception is supported by studies from Sharma et al. and Anwar et al., which found similar results [40, 41]. Systematic reviews suggest that herbal toothpaste is more effective than non-herbal toothpaste at removing supra-gingival plaque with short-term use of 1 month. However, no significant difference was observed between the two types of toothpaste with long-term use of 3 months [16]. Additionally, herbal toothpaste containing specific natural ingredients has been reported to be effective in the reduction of plaque and gingivitis when compared to non-herbal toothpaste [27, 42]. However, its active components’ safety requires evaluating their concentrations and toxicity thresholds [43].

Fluoride-containing toothpastes are well established for caries prevention, primarily by promoting enamel remineralization and reducing demineralization during repeated cariogenic challenges [5–7, 44, 45]. In Thailand, the Dental Association of Thailand recommends toothpaste containing 1400–1500 ppm fluoride for individuals aged 6 years and older, whereas 1000 ppm fluoride is generally recommended for younger children, depending on age and caries risk [46]. Thai regulations also limit the total fluoride concentration in toothpaste to 0.15% w/w, corresponding to approximately 1500 ppm F, as toothpaste is regulated as a cosmetic product under the Cosmetic Act B.E. 2558 (2015) [34]. Notably, at the time the products examined here were purchased and analysed, there was no legal requirement to declare the fluoride concentration on the product label, which is consistent with the present observation that most products provided no information on the fluoride type or concentration. This regulatory gap has since been addressed by the Notification of the Cosmetics Committee on the Label of Toothpaste Containing Fluoride B.E. 2568 (2025), effective 25 December 2025, which requires the label to state that the product contains fluoride and to declare the total fluoride concentration in ppm [47]. The present findings therefore provide baseline evidence from the period before this requirement took effect and support its value for consumers who choose herbal formulations. Accordingly, the fluoride concentrations measured in this study should be interpreted within both the preventive recommendations and the regulatory framework applicable in Thailand. However, most herbal toothpastes examined in this research had absent or low fluoride levels below 600 ppm [6, 48, 49], including 10 out of 15 samples (66.67%). Only three brands contained around 1500 ppm fluoride; two had 1000 ppm, one contained 500 ppm, and another had 100 ppm. The reason why manufacturers do not include fluoride as an ingredient is unclear. The authors speculate that fluoride might alter the consistency and taste of the toothpaste. It is further proposed that fluoride could be excluded from toothpaste formulations to meet consumer demand for natural, biodegradable, and environmentally friendly products free from chemical contaminants [50–52]. This highlights a concern for patients using herbal toothpaste: it may not effectively prevent caries if it does not contain sufficient fluoride. From a caries-prevention perspective, the absence or low concentration of fluoride is the most clinically important shortcoming identified among the products examined, because fluoride remains the ingredient in these formulations with the strongest evidence base for caries prevention [5–7]. Importantly, incorporating fluoride does not appear to compromise the biocompatibility of a herbal formulation. Fluoride concentration was not associated with greater cytotoxicity in the present data; if anything, the opposite was observed, with weak positive correlations between fluoride concentration and the LC50 of L929 cells (rs = 0.322, *p* = 0.031) and of human gingival fibroblasts (rs =  0.267, *p* = 0.076). In the present study, the three products that combined an adequate fluoride concentration with effective antibacterial activity and low cytotoxicity (CUdent Triple Herb, Parodontax Herbal Fresh, and Hi-Herb Day & Night Care) indicate that a herbal formulation and an effective fluoride concentration are not mutually exclusive. Manufacturers of herbal toothpastes should therefore be encouraged to include fluoride at concentrations consistent with national recommendations, and clear labeling should enable consumers who prefer herbal products to identify the formulations that also provide evidence-based caries protection.

The antibacterial efficacy test aims to evaluate the ability of a substance, such as herbal toothpaste, to inhibit or kill bacterial growth. This test is crucial in determining the product’s effectiveness in reducing or eliminating harmful oral bacteria, such as *S. mutans*, which are commonly associated with dental caries and other oral infections. *S. mutans* was selected because of its well-established role in dental caries and its acidogenic, aciduric, and biofilm-forming properties. It is also commonly used as a representative cariogenic microorganism in in vitro antibacterial studies of toothpastes [37]. However, dental caries is a polymicrobial, biofilm-mediated disease, and the antibacterial activity observed against *S. mutans* alone may not fully represent the response of the complex oral microbiota.

By comparing the antibacterial properties of different formulations, researchers can identify the most effective ingredients and optimize the product for better oral health outcomes. The results of this study revealed that Tepthai Concentrated Herbal Toothpaste, Darlie Salt Herbal Protect, Parodontax Herbal Fresh, and Moa Jula Herbal Toothpaste demonstrated potential in inhibiting *S. mutans*, with no statistically significant difference compared to the positive control. A significantly higher zone of inhibition was observed in toothpastes with sodium lauryl sulfate. The high antibacterial efficacy of certain toothpaste formulations can be attributed to their active herbal ingredients. Tepthai Concentrated Herbal Toothpaste, for example, contains guava extract, *Streblus asper* leaf extract, licorice extract, *Eclipta prostrata* extract, peppermint extract, and clove oil, which have been reported in previous publications to possess antibacterial properties and biofilm formation [19, 20, 25, 26]. Similarly, Moa Jula Herbal Toothpaste includes essential oils such as *Mentha piperita* oil (peppermint), *Cinnamomum cassia* leaf oil (cinnamon), and *Eugenia caryophyllus* flower oil, all of which have demonstrated significant antibacterial and anti-inflammatory effects [18].

Parodontax Herbal Fresh also showed strong antibacterial activity against *S. mutans*. This effect may be associated with its overall formulation, including sodium bicarbonate and other active ingredients. Sodium bicarbonate has been reported to contribute to acid neutralization within dental biofilms [53]; however, the specific contribution of individual ingredients to the antibacterial activity observed in the present study cannot be determined. Darlie Salt Herbal Protect, containing sodium monofluorophosphate, sodium bicarbonate, and sodium chloride, exhibited notable antibacterial activity [53, 54]. The antibacterial efficacy of active agents depends on their concentration and amount, which should be indicated in product formulations.

In the aspect of cytotoxicity, oral care products should not be irritating to the gums and other soft tissues. These products must be formulated to be gentle while effectively maintaining oral health. Evaluating the cytotoxicity of herbal toothpastes is crucial to ensure they are safe for daily use and do not harm the delicate tissues of the mouth. This study evaluated cytotoxicity using two cell types: the L929 mouse fibroblast cell line and primary human gingival fibroblasts (HGF). L929 cells are commonly employed in toxicological assays due to their reproducibility and sensitivity, as recommended in ISO 10993-5:2009. In contrast, HGF cells, derived from human oral tissue, better reflect the biological responses of the oral environment to topically applied substances. The results demonstrated a consistent trend across both cell types, with products such as Tepthai Concentrated Herbal Toothpaste and Moa Jula Herbal Toothpaste showing the highest cytotoxicity. At the same time, Goodage Herbal Total Care exhibited the lowest. LC₅₀ values in HGF cells were generally lower than those in L929 cells, indicating higher sensitivity and reinforcing the relevance of using human-derived cells for evaluating oral biocompatibility. These findings align with previous reports, such as Eldeniz et al., who also utilized both L929 and HGF cells to assess the cytotoxicity of dental materials, highlighting the value of a dual-cell approach for more comprehensive safety evaluation [55]. Using standardized and tissue-specific cell models enhances the reliability and clinical relevance of in vitro testing in oral healthcare product development.

This study evaluated the cytotoxic effects of different toothpaste formulations, focusing on identifying specific ingredients that may only influence cell viability in HGF cells. The findings indicate that essential oils such as clove oil (eugenol, *Eugenia caryophyllus*), camphor, menthol, borneol, and peppermint, which are present in Tepthai, Tree Herb, Moa Jula, and Colgate, may contribute to higher cytotoxicity due to their potential to disrupt cell membranes and induce oxidative stress [30]. Additional agents, including aluminum sulfate and zinc compounds, may reduce cell viability [11, 39]. Although sodium lauryl sulfate (SLS), a known cytotoxic agent, was present in these formulations, its effects appear to be enhanced when combined with volatile oils and unmitigated by protective agents [12, 39]. This observation highlights the need for future studies to investigate further the synergistic interactions between SLS and volatile components in oral care products. In contrast, Goodage Herbal Total Care exhibited the lowest cytotoxicity, likely owing to its antioxidant-rich plant extracts (*Phyllanthus emblica* and *Terminalia bellerica*), milder surfactants like sodium cocoyl glutamate, and a balanced preservative system. For instance, Pinmai et al. reported that extracts of *P. emblica* and *T. bellerica* exhibited growth-inhibitory effects on human hepatocellular carcinoma (HepG2) and lung carcinoma (A549) cells and also demonstrated a synergistic effect when combined with doxorubicin or cisplatin [56]. These findings support the potential role of antioxidant-rich extracts in modulating cellular responses and reducing cytotoxicity in HGF cells. The results highlight the importance of formulation balance, particularly the ratio between bioactive and protective compounds, in determining the biocompatibility of herbal toothpaste. However, the mechanisms underlying the interaction between SLS and antioxidant components remain unclear and warrant further investigation, especially concerning the concentration ratios and exposure duration.

Ideal herbal toothpastes should offer antibacterial properties without inducing cytotoxicity, while maintaining an effective fluoride concentration for caries prevention. This study investigated the fluoride content, cytotoxicity to HGF cells, and antibacterial efficacy against *S. mutans* in various herbal toothpaste formulations. The findings revealed that 66.67% of the products (10 of 15) contained less than 600 ppm fluoride. Only three formulations (20%) had high fluoride levels within the range of 1500 ppm. Additionally, most herbal toothpastes did not specify the fluoride type or concentration on their labels. Only two products (13.33%) accurately stated both parameters, and their measured values were consistent with the labeled information. Antibacterial activity and cytotoxicity appeared to be influenced by other active ingredients in the formulations. Products with strong antibacterial activity often exhibited moderate to high cytotoxicity, likely due to essential oils such as clove oil, borneol, and camphor, which possess antimicrobial properties but may increase cytotoxic effects, especially when combined with surfactants like sodium lauryl sulfate (SLS). In contrast, formulations that incorporate biocompatible components—such as antioxidants and mild surfactants—may offer a more favorable balance between antimicrobial efficacy and cellular safety. Among the tested products, CUdent Triple Herb, Parodontax Herbal Fresh, and Hi-Herb Day & Night Care demonstrated the most ideal characteristics, as they exhibited high fluoride concentrations, effective antibacterial activity, and low cytotoxicity. These beneficial characteristics are likely contributed by ingredients such as *Mentha piperita* (menthol) and *Eugenia caryophyllus* (eugenol) present in CUdent Triple Herb and Hi-Herb Day & Night Care, as well as the exclusion of SLS in CUdent Triple Herb and Parodontax Herbal Fresh.

This study is limited by its reliance on in vitro assessments, which may not fully reflect real-life oral health conditions. While the findings offer valuable preliminary insights, they do not account for individual variations, daily habits, or long-term effects that require clinical validation. The antibacterial assessment was limited to *S. mutans* and therefore did not fully represent the polymicrobial nature of cariogenic biofilms. A non-selective Mueller–Hinton agar supplemented with 5% sheep blood was used, although the inoculum was prepared from a pure *S. mutans* culture. In addition, the antibacterial assay was conducted as a single experiment with three replicate disks per formulation; biological replication across independent experiments was therefore not performed, and the number of replicates was limited. These limitations should be considered when interpreting the antibacterial findings. Future studies should include additional cariogenic species, multispecies biofilm models, and a larger number of replicates to better represent the oral environment.

To ensure the safety and efficacy of herbal toothpastes, future research should involve well-designed clinical trials that assess product performance under typical usage. These studies would also help evaluate patient compliance and satisfaction, contributing to more effective product development.

## Conclusions

The herbal toothpastes evaluated in this study exhibited varying levels of fluoride, with most formulations containing low concentrations. Despite this, strong antibacterial activity was observed, likely due to the presence of essential oils and other active ingredients, such as sodium lauryl sulfate (SLS). However, formulations with higher antibacterial efficacy tended to also show higher cytotoxicity, a relationship supported by the inverse correlation between the zone of inhibition and the LC50 of L929 cells (rs = **–**0.579, *p* < 0.001) and of human gingival fibroblasts (rs = **−**0.328, *p* = 0.028). Products whose formulations contained antioxidant-rich extracts were among those with the lowest cytotoxicity; antioxidant content was not quantified in this study, so this observation was not tested statistically. These findings suggest that the antibacterial effects of herbal toothpastes may stem from active compounds other than fluoride, but caution is needed when interpreting these results in clinical settings. Further clinical studies are necessary to assess the long-term safety and effectiveness of these products.

## Data Availability

The datasets used and/or analyzed during the current study are available from the corresponding author on reasonable request.
